# Association between quality and quantity of carbohydrate intake with selected anthropometric indices among primary school girls in Kerman city, Iran: a cross-sectional study

**DOI:** 10.1186/s12887-024-04739-6

**Published:** 2024-04-24

**Authors:** Nooshin Jannati, Reyhaneh Mohammadi-Faez, Mohammad Reza Mahmoodi, Leila Azadbakht

**Affiliations:** 1 Department of Community Nutrition, School of Nutritional Sciences and Dietetics, Tehran University of Medical Sciences (TUMS), Tehran, Iran; 2 Department of Nutrition, Faculty of Public Health, Kerman University of Medical Sciences, Kerman, Iran; 3 Physiology Research Center, Institute of Neuropharmacology & Department of Nutrition, Faculty of Public Health, Kerman University of Medical Sciences, Kerman, Iran

**Keywords:** Quality, Quantity, Carbohydrate, Anthropometric indices, Student, Kerman

## Abstract

**Background:**

The school-age is a crucial period of physical and cognitive growth, which requires an assessment of dietary intake and its influence on body weight and height. This study aims to determine the association between the quality and quantity of carbohydrate intake with selected anthropometric indices in primary school girls in Kerman City, Iran.

**Methods:**

This cross-sectional study was conducted on 330 primary school girls ages 6–12 years in Kerman. We used a validated and reliable dish-based 185-item food frequency questionnaire to evaluate people’s food intake. We assessed the amount of carbohydrates in children’s diets as the percentage of daily calories and grams per day. We used dietary fiber intake (grams/day), the glycemic load, the ratio of whole grain to total grain, and the ratio of solid carbohydrates to total carbohydrates to assess carbohydrate quality. Height, weight, and arm circumference were measured. We calculated body mass index (BMI) by dividing the weight in kilograms by the height in centimeters squared. We used the World Health Organization z-scores charts for girls 5 to 19 years old to assess z-scores for BMI-for-age (BAZ), height-for-age (HAZ), and weight-for-age (WAZ). Socioeconomic status and physical activity were assessed. We used ANOVA and ANCOVA statistical tests to assess the association between anthropometric indices and carbohydrate quantity and quality parameters.

**Results:**

Participants with the highest amount of carbohydrate intake had significantly higher anthropometric indices, including arm circumference, BMI-for-age z score (BAZ), and Height-for-age z score (HAZ) (*p* < 0.001, *p* < 0.001, and *p* = 0.009, respectively). With the increase in glycemic load (GL) and dietary fiber intake, anthropometric indices including arm circumference (GL: *p* < 0.001, Fiber: *p* < 0.001), BAZ (GL: *p* < 0.001, Fiber: *P* < 0.001), and HAZ (GL: *P* = 0.009, Fiber: *p* < 0.001) increased significantly.

**Conclusions:**

We concluded that there was a positive association between the amount of carbohydrate intake and anthropometric indices (MUAC, BAZ, and HAZ). Also, with the increase in glycemic load and dietary fiber intake, the anthropometric indices including MUAC, BAZ, and HAZ increased.

## Background

Child growth is a critical indicator of society’s nutrition and health status [1]. ‌‌‌‌‌Anthropometry parameters, including body weight, height, and body mass index (BMI), describe the growth pattern in children and adolescents [2]. Weight disorders and stunting in childhood and adolescence are prevalent in Asians [1, 3, 4]. The children malnutrition (undernutrition) rate in Kerman based on weight-for-age (under-weight), height-for-age (stunting), and weight-for-height (thinness, wasting) are 6.06, 5.58, and 75.9%, respectively, and malnutrition is higher in girls than boys [5]. Childhood obesity is potentially critical for adulthood disease, including metabolic syndrome, type 2 diabetes, coronary artery disease, non-alcoholic fatty liver disease, and some cancers [6].

On the other hand, being underweight increases the risk of infectious diseases in children and adolescents; and stunting can affect growth by reducing efficiency due to muscle loss and increasing the risk of childbirth in adulthood period [7, 8]. Therefore, children’s diagnosis and treatment of growth disorders should be considered early in life. Based on the existing literature, nutrition plays a crucial role in the growth of every child and affects their later development of chronic diseases [9].

With regards to diet, a healthy diet in childhood is critical for children’s health and well-being and forms lifelong eating habits [10]. Globally, children’s diets are poor. The characterization of their current diet is insufficient consumption of foods such as vegetables and whole grains and excessive consumption of nutrient-poor foods and beverages [11]. In a systematic evaluation study it was reported that low diet quality had an inverse relationship with risk factors for chronic diseases such as overweight and less/unfavorable child growth outcomes [12]. Therefore, checking the quality of children’s diets is critical. Studies have shown that the quantity of each macronutrient, such as carbohydrates, proteins, and fats, affects body weight, and the type and quality of foods have different effects [13].

A cross-sectional study on Iranian children concluded that lower carbohydrate intake increases the odds of being underweight [14]. However, in a study on children aged 1–10, no association was found between the intake of carbohydrates and body mass index or body composition in children [15]. In addition to the amount of total food carbohydrates, different types of carbohydrates and their sources should also be considered. An approach to define carbohydrate quality involves utilizing “carbohydrate quality index (CQI)” that includes four characteristics of carbohydrate quality (The total dietary fiber intake (grams/day), the glycemic load, the ratio of whole grain to total grain and the ratio of solid carbohydrates to total carbohydrates) [16]. A study on Iranian adults showed an inverse association between CQI and body weight [17]. However, no association was found between the dietary carbohydrate indices and general and central obesity in Iranian women [18].

The vulnerability of children necessitates the monitoring of macronutrient intake quantity and quality to assess diet quality and its correlation with anthropometric indices. Meanwhile, the nutrition and health of girls are doubly important because they are future mothers and play a significant role in the health and nutrition of children. To our knowledge, this is the first study that evaluates the association between the quality and quantity of carbohydrate intake with selected anthropometric indices among primary school girls in Kerman city.

## Methods

### Study design and subjects

This cross-sectional study was conducted on 330 primary school girls in Kerman, Iran, using cluster random sampling methods. Kerman province is one of the 31 provinces of Iran. Kerman is in the southeast of Iran with its administrative center in Kerman City. It is the first largest province of Iran that encompasses nearly 11% of the land area of Iran. The province’s population is about 3 million (9th in the country). To calculate the required sample size in this study, the mean and standard deviation of BMI from a cross-sectional study conducted on 7-11-year-old Iranian children were used (Mean ± SD = 16.0 ± 2.9 kg/m²) [19]. Then, the sample size was calculated using the following formula:$n = \left[ {{{\left( {{z_1} - \alpha {/_2}} \right)}^2} \times {s^2}} \right]/{d^2}$with d = 2% and alpha = 0.05, the calculated sample size is 323. In order to strengthen the study, the obtained number was estimated to be 330. Power of the study was 80%. Participants who entered this study met the following criteria: (1) willingness to participate (completed a consent form); (2) those between 6 and 12 years old; (3) absence of chronic diseases including diabetes, congenital metabolic diseases such as maple syrup urine disease, and phenylketonuria, thyroid gland diseases, epilepsy, and asthma; and (4) no use of corticosteroids, thyroid medications, diabetes medications, epilepsy medications, or allergy medications. We excluded children whose parents did not complete the consent form. The Ethics Committee of Tehran University of Medical Sciences approved the study protocol (IR.TUMS.MEDICINE.REC.1400.582). All guardians completed the written consent form.

### Data collection tool

We collected all data using reliable and validated questionnaires, including the questionnaire on socioeconomic status, the International Physical Activity Questionnaire short form [20], and a dish-based food frequency questionnaire (FFQ). All data was gathered by interviewing the children’s parents.

### Dietary intake

We designed a dish-based food frequency questionnaire to evaluate the nutritional status of participants. Then, the researchers tested the validity and reliability of the questionnaire (presented in Sect. 2.8). The children’s parents completed this questionnaire. The frequency of children’s consumption was determined by parents according to their consumption of foods in the last year on a daily, weekly, or monthly basis. We used the manual of household scales to convert the amount mentioned for each food item to grams. Dietary intakes were analyzed using the NUTRITIONIST IV software (First Data Bank, San Bruno, California) to estimate energy and nutrient intake. Finally, we entered the data into SPSS for statistical analysis.

### Demographic and socioeconomic status

Demographic and Socioeconomic Status (SES) was assessed using a valid and reliable questionnaire developed for estimating SES and its relationship with health outcomes among Iranians. Questions on the education and job of parents, number of family members, house ownership or tenancy, car ownership, number of cars, number of bedrooms in the house, and having appliances such as washing machine, dishwasher, LCD TV, side-by-side refrigerator, air conditioner, vacuum cleaner, computer, laptop, and advanced heating system were included in this questionnaire [21]. Each questionnaire item was encoded to calculate the socioeconomic status score. Finally, the codes were added up and divided into three groups for qualitative description: weak, medium, and rich.

### Anthropometric indices

Children’s body weight, height, and mid-upper arm circumference (MUAC) were measured. We used a digital scale with a precision of 100 g to measure body weight with minimal clothing and no shoes. A plastic tape attached to the wall at 0.1 cm precision was used to measure height. Body weight (kilograms) divided by the square height (in square meters) to calculate BMI. MUAC was measured using tape at the point between the shoulder and elbow. The guidelines of the World Health Organization [22] to calculate z-scores for BMI-for-age (BAZ), height-for-age (HAZ), and weight-for-age (WAZ), were used. Thus, BAZ was categorized into obese (BAZ ≥ 2 SD), overweight (BAZ ≥ 1 SD), normal (BAZ ≥ − 1 SD and < − 1 SD), and underweight (BAZ < − 2 SD). Categories for HAZ are: short stature (HAZ ≤ − 2 SD) and severe short stature (HAZ ≤ − 3 SD). Categories for WAZ: -3 SD < WAZ <-1 SD considered underweight, and WAZ ≤ − 2 SD considered severely underweight.

### Physical activity

Physical activity assessment was done using the International Physical Activity Questionnaire (IPAQ) short form [20]. Scores were calculated according to the frequency and time spent on light, moderate, and high activities and sedentary periods over the last seven days. Physical activity (PA) levels are expressed as the metabolic equivalent hours per week. PA level was categorized into: low physical activity (< 600 METs-min/week); moderate physical activity (between 600 and 3,000 METs-min/week); and high physical activity (> 3,000 METs-min/week).

### Quality and quantity of carbohydrate calculation

The amount of carbohydrates in children’s diets was calculated as a percentage of daily calories and grams/day. The total dietary fiber intake (grams/day), the glycemic load, the ratio of whole grain to total grain and the ratio of solid carbohydrates to total carbohydrates were used (solid carbohydrates + liquid carbohydrates) for assessing carbohydrate quality [16]. We used the formula to calculate total dietary GI: ∑ (GI_a_ × available carbohydrates_a_)/total available carbohydrates, where available carbohydrates were calculated as total carbohydrates_a_ minus fiber [23]. GI values were extracted from the Iranian GI table [24] and the international table [25]. For foods that were not in the tables, the mean of the same foods in the FFQ was calculated [26]. In addition to the glycemic index of a diet, the glycemic load is a measurement of both the quality and quantity of carbohydrates consumed, taking into account both the GI (the impact on blood glucose levels) and the amount of carbohydrates ingested. The dietary GL was calculated using the formula: (total GI × total available carbohydrate)/100 [23]. We categorized carbohydrate intake by physical form: liquid carbohydrate intake included sugar-sweetened beverages and fruit juices; Solid carbohydrate intake included all solid carbohydrate foods [27].

### Validity and reliability of food frequency questionnaire

In this study, to determine the reproducibility, the questionnaire was completed by 56 parents 12 weeks apart and measured using the intra-class correlation coefficient. Three 24-hour recalls were collected during the study. Pearson correlations and the Wilcoxon signed-rank test were used to assess validity. The strength of the relationship for validity and reliability data using the following correlation rating interpretations were accomplished: Pearson statistics—0.10 to 0.30 weak, 0.30 to 0.50 moderate, > 0.50 strong [28]. ICC statistics—0.00 to 0.10, virtually none, 0.11 to 0.40 slight, 0.41 to 0.60 fair, 0.61 to 0.80 moderate, and 0.81 to 1.0 substantial [29].

### Statistical analysis

The normal distribution of all variables was checked using the histogram and Kolmogorov-Smirnov test. The logarithmic equivalent (Ln transformation) was used for the variables that do not have a normal distribution in the analyses. We used one-way ANOVA to evaluate the association between carbohydrates intake as independent variables (i.e., amount of carbohydrates, dietary fiber intake (grams per day), the glycemic load, the ratio of whole grain to total grain and the ratio of solid carbohydrates to total carbohydrates (solid carbohydrates + liquid carbohydrates)) and anthropometric indices as dependent variables. The chi-square test was used for descriptive characteristics (i.e., supplement use, socioeconomic level, etc.) to compare how individuals were distributed throughout the groups. We also used an analysis of covariance to adjust the possible effects of all potentially confounding variables. We entered variables including age supplement use, parent smoking, physical activity, and socioeconomic status into the adjustment model. Binary logistic regression was used to determine the odds ratios (ORs) and 95% confidence intervals (CIs) for the presence of underweight and overweight/ obesity across Qs of carbohydrate quantity and quality in unadjusted and adjusted models. In the crude and adjusted model, first tertile was considered the reference group, and the odds ratio of other tertiles was calculated. Data were presented as mean ± standard deviation (Standard Error), odds ratio, and 95% confidence interval. SPSS software (22.0; SPSS Inc.) was used for all statistical analyses, and a p-value < 0.05 was considered statistically significant.

## Results

### Participant characteristics

Table 1 represents the sociodemographic characteristics of the participants. The participants’ mean age was 9.02 ± 1.813 years, and their mean BMI was 17.49 ± 3.93 kg/m^2^. The frequency of supplement use in 330 participants was 18.5%. Three hundred-one participants (91.2%) mainly reported low levels of physical activity, with only 0.3% classified as having high physical activity. 56.4% of participants (*n* = 186) were in the low socioeconomic status group.

**Table 1:** Characteristics of participants (primary school girls in Kerman) in tertiles of carbohydrates quantity

Tertiles of carbohydrate quantity (g)					
Variable		Tertile 1 ≤ 218.7 *N* = 111	Tertile 2 > 218.7 < 287.1 *N* = 109	Tertile 3 ≥287.1 *N* = 110	*p* value*
**Quantitative variables/ mean ± SD**					
Age (year) Body weight (kg) Height (cm) BMI (kg/m2) MUAC (cm) Physical activity (met/min/week)		8.73 (1.78) 28.77 (10.72) 132.62 (12.77) 15.89 (3.30) 20.49 (3.1) 143.58 (328.77)	9.07 (1.76) 34.97 (12.41) 138.09 (13.62) 17.76 (3.49) 22.18 (3.46) 129.33 (257.39)	9.26 (1.85) 37.74 (14.19) 139.38 (13.45) 18.83 (4.36) 23.26 (4.03) 308.58 (599.12)	0.085 0.0001 0.0001 0.0001 0.0001 0.002
Qualitative variables /N (%)					
Grade	1st 2nd 3rd 4th 5th 6th	23 (7%) 26 (7.9%) 20 (6.1%) 12 (3.6%) 15 (4.5%) 15 (4.5%)	17 (5.2%) 11 (3.3%) 20 (6.1%) 22 (6.4%) 20 (6.1%) 19 (5.8%)	18 (5.5%) 14 (4.2%) 15 (4.5%) 19 (5.8%) 22 (6.7%) 22 (6.7%)	0.132
Socioeconomic status	Low Medium High	83 (25.2%) 28 (0.85%) 0 (0.0%)	54 (16.4%) 54 (16.4%) 1 (0.3%)	49 (14.8%) 55 (16.7%) 6 (1.8%)	0.0001
Supplement	Yes No	14 (4.2%) 97 (29.4%)	18 (5.5%) 91 (27.6%)	28 (8.5%) 82 (24.8%)	0.04
Parents smoking	Yes No	9 (2.7%) 102 (30.9%)	11 (3.3%) 98 (29.7%)	18 (5.5%) 92 (27.9%)	0.134
Physical activity	Low Medium High	106 (32.1%) 5 (1.5%) 0 (0.0%)	103 (31.2%) 6 (1.8%) 0 (0.0%)	92 (27.9%) 17 (5.2%) 1 (0.3%)	0.014
Fathers’ education	High school Diploma College	27 (8.2%) 58 (17.6%) 26 (7.9%)	15 (4.5%) 55 (16.7%) 39 (11.8%)	17 (5.2%) 49 (14.8%) 44 (13.3%)	0.046
Mothers’ education	High school Diploma College	24 (7.3%) 65 (19.7%) 22 (6.7%)	10 (3.0%) 57 (17.3%) 42 (12.7%)	9 (2.7%) 54 (16.4%) 47 (14.2%)	0.0001
Family member	4 or less 5 to 7 More than 7	70 (21.2%) 39 (11.8%) 2 (0.6%)	75 (22.7%) 32 (9.7%) 2 (0.6%)	83 (25.2%) 25 (7.6%) 2 (0.6%)	0.385
Fathers’ occupation	Manager Employee Worker Homemaker Retired Self-employment Others	3 (0.9%) 24 (7.3%) 18 (5.5%) 0 (0.0%) 0 (0.0%) 63 (19.1%) 3 (0.9%)	2 (0.6%) 33 (10.0%) 6 (1.8%) 1 (0.3%) 1 (0.3%) 65 (19.7%) 1 (0.3%)	3 (0.9%) 36 (10.9%) 6 (1.8%) 0 (0.0%) 2 (0.6%) 61 (18.5%) 2 (0.6%)	0.134
Mothers’ occupation	Manager Employee Worker Homemaker Retired Self-employment Others	0 (0.0%) 10 (3.0%) 1 (0.3%) 92 (27.9%) 0 (0.0%) 6 (1.8%) 1 (0.3%)	2 (0.6%) 14 (4.2%) 0 (0.0%) 85 (25.8%) 0 (0.0%) 6 (1.8%) 3 (0.9%)	0 (0.0%) 7 (2.1%) 0 (0.0%) 88 (26.7%) 1 (0.3%) 12 (3.6%) 2 (0.6%)	0.260

*The *P* value reported for the quantitative variables was resulted from one-way ANOVA, and the numbers are reported as mean ± SD. *The *P* value for the qualitative variables was calculated by the chi-square test, and the results are based on N (%). *P value < 0.05 shows a significant level of association

### Validity and reliability of food frequency questionnaire

The findings of this study showed that the developed FFQ is reliable and valid. Correlation coefficients between dietary intake estimates derived from FFQ and 24-HRs were 0.52 for carbohydrates, 0.54 for proteins, and 0.51 for fat. Furthermore, the Wilcoxon signed-rank test did not show significant differences between FFQ and 3DR for most of the nutrients (*p* > 0.05). Intraclass correlation coefficients used to measure the reproducibility of FFQ ranged from 0.54 to 0.77 (Table 2). This FFQ is a helpful tool in dietary assessment in this age group.

**Table 2:** Reproducibility and validation study: Correlation coefficient and Wilcoxon signed-rank test for validity and ICC for reproducibility

	FFQ	3DR^4^	Wilcoxon signed rank test (p Value)^1^	Correlation coefficient^2^	ICC^3^
	Mean ± SD	Mean ± SD			
Carbohydrate (g)	262.22(92.28)	235.75(84.97)	0.013	0.7	0.52
Protein (g)	63.42(22.62)	58.75(20.94)	0.059	0.63	0.54
Fat (g)	64.02(25.76)	60.28(24.58)	0.136	0.61	0.51
Calcium (mg)	748.58(335.10)	696.48(272.37)	0.434	0.54	0.59
Fe (mg)	13.65(5.01)	12.77(4.78)	0.016	0.75	0.74
Magnesium (mg)	218.52(76.82)	201.07(81.82)	0.013	0.65	0.60
Vitamin C (mg)	97.48(54.55)	95.75(62.34)	0.211	0.77	0.62
Vitamin A (µg)	777.25(503.65)	745.43(532.87)	0.915	0.68	0.70
Vegetables (g)	170.65(87.34)	175.79(107.75)	0.866	0.59	0.75
Fruits (g)	280.38(155.49)	284.13(170.27)	0.135	0.68	0.65
Meat and its products (g)	89.56(41.06)	78.41(34.25)	0.124	0.68	0.75
Dairy products (g)	394.29(224.29)	370.29(192.16)	0.541	0.89	0.62

^1^ Wilcoxon signed-rank test were used to examine the difference between FFQ and 3DR with a significant *p* value < 0.001 ^2^Pearson or Spearman correlation coefficient were used to assess the correlation between variables with a *p*-value < 0.05 considered as significant. ^3^the intra-class coefficient for the comparison between FFQ1 and FFQ2. ^4^3DR, dietary recall

### Dietary intake of participants

The average consumption of energy, macronutrients, fatty acids, vitamins, minerals, and food groups across the three tertiles of macronutrient consumption is shown in Table 3. All these intakes (except energy intake) were adjusted for energy intake. Intake of energy (2531.12 ± 460.8 vs. 1202.21 ± 277.84 kcal; *p* = 0.0001), potassium (2931.81 ± 61.91 vs. 2650.47 ± 59.51 mg; *p* = 0.016), dairy products (441.06 ± 26.21 vs. 330.89 ± 25.19 g; *p* = 0.021), grains (148.46 ± 6.33 vs. 97.15 ± 6.09 g; *p* = 0.0001), fruits (331.23 ± 17.73 vs. 241.77 ± 17.05 g; *p* = 0.009), and vitamin A (865.83 ± 67.83 vs. 589.61 ± 65.19 µg; *p* = 0.033) was higher in the highest tertile of carbohydrate intake compared to the lowest. The amount of MUFA (18.27 ± 0.475 vs. 20.08 ± 0.456 g; *p* = 0.027), PUFA (12.87 ± 0.447 vs. 14.94 ± 0.49 g; *p* = 0.029), zinc (7.81 ± 0.17 vs. 8.22 ± 0.16 mg; *p* = 0.047), and selenium (0.091 ± 0.003 vs. 0.108 ± 0.003 mg; *p* = 0.003) decreased significantly in the higher tertiles of carbohydrate intake.

**Table 3:** Dietary intake of participants (primary school girls in Kerman) in tertiles of carbohydrate intake

Tertiles of carbohydrate quantity				
Variable	Tertile 1 ≤ 218.7 *N* = 111	Tertile 2 > 218.7 < 287.1 *N* = 109	Tertile 3 ≥287.1 *N* = 110	*p* value*
Mean ± SD				
Energy (kcal/d) Fiber (g/d) Cholesterol (mg/d) SFA (g/d) MUFA (g/d) PUFA (g/d)	1202.21 (277.84) 13.61 (0.35) 265.06 (9.25) 23.06 (0.90) 20.08 (0.45) 14.94 (0.42)	1806.88 (193.05) 14.07 (0.23) 268.38 (6.19) 21.9 (0.60) 19.82 (0.30) 14.08 (0.28)	2531.12 (460.80) 14.77 (0.37) 251.48 (9.63) 19.66 (0.94) 18.27 (0.47) 12.87 (0.44)	< 0.001 0.195 0.319 0.103 0.027 0.029
Vitamin A (µg) Vitamin D (µg) Vitamin E (mg) Vitamin K (mg) Thiamine (mg) Riboflavin (mg) Niacin (mg) Vitamin B5 (mg) Vitamin B6 (mg) Folic acid (µg) Vitamin B12 (µg) Vitamin C (mg)	589.61 (65.19) 1.12 (0.14) 12.42 (0.40) 173.37 (6.63) 1.61 (0.03) 1.41 (0.04) 16.24 (0.27) 5.06 (0.10) 1.45 (0.05) 218.11 (6.14) 3.29 (0.11) 91.83 (6.02)	782.61 (43.61) 1.49 (0.09) 11.76 (0.27) 164.09 (4.43) 1.62 (0.02) 1.52 (0.02) 16.07 (0.18) 5.15 (0.07) 1.51 (0.03) 213.64 (4.11) 3.43 (0.07) 93.35 (4.03)	865.83 (67.83) 1.66 (0.15) 10.98 (0.42) 160.8 (6.90) 1.63 (0.03) 1.52 (0.04) 16.25 (0.28) 4.93 (0.11) 1.51 (0.05) 208.4 (6.39) 3.22 (0.12) 107.25 (6.27)	0.033 0.075 0.151 0.475 0.908 0.06 0.751 0.210 0.647 0.684 0.198 0.192
Calcium (mg) Magnesium (mg) Potassium (mg) Zinc (mg) Fe (mg) Phosphorus (mg) Selenium (mg)	682.55 (30.37) 214.95 (4.06) 2650.47 (59.5) 8.22 (0.16) 13.22 (0.27) 1055.05 (24.23) 0.108 (0.003)	767.34 (20.32) 222.01 (2.72) 2841.52 (39.8) 8.31 (0.11) 13.55 (0.18) 1098.76 (16.21) 0.103 (0.002)	796.60 (31.60) 218.67 (4.23) 2931.81 (61.91) 7.81 (0.17) 14.18 (0.28) 1063.23 (25.21) 0.091 (0.003)	0.053 0.246 0.016 0.047 0.129 0.130 0.003
Grains (g) Fruits (g) Vegetables (g) Meat and its products (g) Beans (g) Nuts and seeds (g) Dairy products (g) Fats (g) Added sugar beverages (g) Chocolate and snacks (g)	97.15 (6.09) 241.77 (17.05) 270.79 (10.02) 82.55 (3.70) 39.46 (2.08) 16.66 (1.68) 330.89 (25.19) 12.30 (0.53) 69.09 (11.68) 202.73 (18.85)	113.34 (4.12) 268.15 (11.50) 273.57 (6.70) 80.17 (2.47) 36.47 (1.39) 14.96 (1.12) 411.65 (16.85) 12.81 (0.35) 81.69 (7.81) 210.95 (12.77)	148.46 (6.33) 331.23 (17.73) 286.74 (10.42) 72.48 (3.85) 33.80 (2.17) 13.07 (1.74) 441.06 (26.21) 11.91 (0.55) 111.89 (12.16) 170.83 (19.69)	< 0.001 0.009 0.579 0.244 0.321 0.499 0.021 0.267 0.098 0.216

SFA = saturated fatty acids, MUFA = monounsaturated fatty acids, and PUFA = polyunsaturated fatty acids. *The *p* value is reported from covariance analysis, and the results are based on mean ± SD. ∗All of the variables are adjusted for energy intake. *p value < 0.05 shows a significant level of association

### Anthropometric indices across tertiles of carbohydrate quantity

There was a significant positive association between carbohydrate consumption (gram) and anthropometric indices, including arm circumference (23.01 ± 0.29 vs. 20.82 ± 0.29 cm; *p* < 0.001), BAZ (0.64 ± 0.13 vs. -0.43 ± 0.13; *p* < 0.001) and HAZ (0.75 ± 0.11 vs. 0.29 ± 0.11; *p* = 0.009). Tertile 3 in compare with tertile 1 was indicated. The adjustment model showed no association between carbohydrate intake and WAZ (*p* = 0.691) (Table 4).

**Table 4:** Association between anthropometric indices and carbohydrate quantity among primary school girls in Kerman

Tertiles of carbohydrate quantity (g)					
Variable		Tertile 1 ≤ 218.7 *N* = 111	Tertile 2 > 218.7 < 287.1 *N* = 109	Tertile 3 ≥287.1 *N* = 110	*p* value*
MUAC (cm)	Model 1 Model 2	20.49 (3.1) 20.82 (0.29)	22.18 (3.46) 22.10 (0.28)	23.26 (4.03) 23.01 (0.29)	< 0.001 < 0.001
BAZ	Model 1 Model 2	-0.49 (1.39) -0.43 (0.13)	0.38 (1.25) 0.35 (0.13)	0.67 (1.45) 0.64 (0.13)	< 0.001 < 0.001
HAZ	Model 1 Model 2	0.23 (1.39) 0.29 (0.11)	0.75 (1.02) 0.73 (0.11)	0.78 (1.16) 0.75 (0.11)	0.001 0.009
WAZ	Model 1 Model 2	-0.22 (1.25) 0.15 (0.91)	0.47 (1.19) 0.34 (0.93)	-0.65 (14.32) -0.79 (0.97)	0.682 0.691

The model 1 was resulted from one-way ANOVA, and the numbers are reported as mean ± SD. Model 2 was resulted from covariance analysis, and the numbers are reported as mean ± SE. *p value < 0.05 shows a significant level of association. Model 1: crude. Model 2: Adjusted for age, supplement use, parents’ smoking, physical activity, and socio-economic status

### Anthropometric indices across tertiles of carbohydrate quality

After adjusting the confounding variables, the participants in the highest tertiles of glycemic load and dietary fiber intake had significantly higher anthropometric indices arm circumference (GL: 22.75 ± 0.29 vs. 20.93 ± 0.29 cm; *p* < 0.001; Fiber: 22.89 ± 0.29 vs. 20.70 ± 0.29 cm; *p* < 0.001), BAZ (GL: 0.57 ± 0.13 vs. -0.38 ± 0.13; *p* < 0.001; Fiber: 0.63 ± 0.13 vs. -0.44 ± 0.13; *p* < 0.001), and HAZ (GL: 0.72 ± 0.11 vs. 0.29 ± 0.11; *p* = 0.009; Fiber: 0.62 ± 0.11 vs. 0.19 ± 0.11; *p* < 0.001). The association between the ratio of whole grain to total grain and anthropometric indices was not significant (*p* ≥ 0.05). No association was observed between the levels of carbohydrate quality indicators and the WAZ of individuals (*p* ≥ 0.05) (Tables 5 and 6).

**Table 5:** Association between anthropometric indices and carbohydrate quality (glycemic load and dietary fiber) among primary school girls in Kerman

Tertiles of carbohydrate quality									
Tertiles of glycemic load					Tertiles of dietary fiber (g)				
Variable		Tertile 1 ≤ 119.94 *N* = 109	Tertile 2 > 119.94 < 155.81 *N* = 111	Tertile 3 ≥155.81 *N* = 110	*p* value*	Tertile 1 ≤ 11.78 *N* = 109	Tertile 2 > 11.78 < 15.62 *N* = 110	Tertile 3 ≥15.62 *N* = 111	*p* value*
MUAC (cm)	Model 1 Model 2	20.52 (3.05) 20.93 (0.29)	22.30 (3.84) 22.22 (0.28)	23.08 (3.77) 22.75 (0.29)	< 0.001 < 0.001	20.37 (3.00) 20.70 (0.29)	22.25 (3.35) 22.30 (0.28)	23.27 (4.14) 22.89 (0.29)	< 0.001 < 0.001
BAZ	Model 1 Model 2	-0.45 (1.39) -0.38 (0.13)	0.37 (1.33) 0.35 (0.12)	0.62 (1.42) 0.57 (0.13)	< 0.001 < 0.001	-0.51 (1.30) -0.44 (0.13)	0.36 (1.41) 0.34 (0.12)	0.69 (1.37) 0.63 (0.13)	< 0.001 < 0.001
HAZ	Model 1 Model 2	0.23 (1.39) 0.29 (0.11)	0.75 (1.05) 0.75 (0.11)	0.78 (1.14) 0.72 (0.11)	< 0.001 0.009	0.12 (1.27) 0.19 (0.11)	0.97 (1.14) 0.95 (0.11)	0.66 (1.11) 0.62 (0.11)	< 0.001 < 0.001
WAZ	Model 1 Model 2	-0.20 (1.26) 0.05 (0.91)	0.50 (1.29) 0.45 (0.91)	-0.73 (14.40) -0.97 (0.98)	0.629 0.563	-0.36 (1.10) -0.082 (0.91)	0.72 (1.32) 0.65 (0.89)	-0.86 (14.68) -1.11 (1.00)	0.445 0.429

The model 1 was resulted from one-way ANOVA, and the numbers are reported as mean ± SD. Model 2 was resulted from covariance analysis, and the numbers are reported as mean ± SE. *p value < 0.05 shows a significant level of association. Model 1: crude. Model 2: Adjusted for age, supplement use, parents’ smoking, physical activity, and socio-economic status

**Table 6:** Association between anthropometric indices and carbohydrate quality (whole grains/total grains and solid carbohydrates/total carbohydrates) among primary school girls in Kerman

Tertiles of carbohydrate quality									
Tertiles of whole grains/total grains						Tertiles of solid carbohydrates/total carbohydrates			
Variable		Tertile 1 ≤0.08 *N* = 109	Tertile 2 > 0.08 < 0.17 *N* = 110	Tertile 3 ≥0.17 *N* = 111	*p* value*	Tertile 1 ≤ 0.59 *N* = 105	Tertile 2 > 0.59 < 0.78 *N* = 112	Tertile 3 ≥ 0.78 *N* = 113	*p* value*
MUAC (cm)	Model 1 Model 2	21.88 (3.86) 21.79 (0.29)	21.40 (3.65) 21.74 (0.29)	22.63 (3.57) 22.38 (0.29)	0.048 0.241	22.12 (3.84) 22.37 (0.30)	22.23 (3.78) 22.09 (0.29)	21.58 (3.53) 21.49 (0.29)	0.379 0.101
BAZ	Model 1 Model 2	0.12 (1.46) 0.11 (0.13)	-0.007 (1.53) 0.04 (0.13)	0.43 (1.33) 0.39 (0.13)	0.071 0.162	0.44 (1.36) 0.49 (0.13)	0.17 (1.52) 0.15 (0.13)	-0.04 (1.44) -0.06 (0.13)	0.046 0.015
HAZ	Model 1 Model 2	0.47 (1.19) 0.46 (0.11)	0.62 (1.34) 0.65 (0.11)	0.66 (1.14) 0.65 (0.11)	0.492 0.421	0.74 (1.12) 0.74 (0.11)	0.55 (1.28) 0.56 (0.11)	0.48 (1.26) 0.47 (0.11)	0.291 0.243
WAZ	Model 1 Model 2	0.13 (1.33) -0.008 (0.94)	-1.02 (13.28) -1.00 (0.87)	0.62 (1.31) 0.73 (0.93)	0.399 0.395	0.66 (1.38) 0.62 (0.91)	-1.13 (13.66) -1.02 (0.91)	0.11 (1.29) 0.03 (0.93)	0.342 0.442

The model 1 was resulted from one-way ANOVA, and the numbers are reported as mean ± SD. Model 2 was resulted from covariance analysis, and the numbers are reported as mean ± SE. *p value < 0.05 shows a significant level of association. Model 1: crude. Model 2: Adjusted for age, supplement use, parents’ smoking, physical activity, and socio-economic status

### Odds ratio and 95% confidence interval for weight disorders in tertiles of carbohydrate quantity

**Fig. 1:**
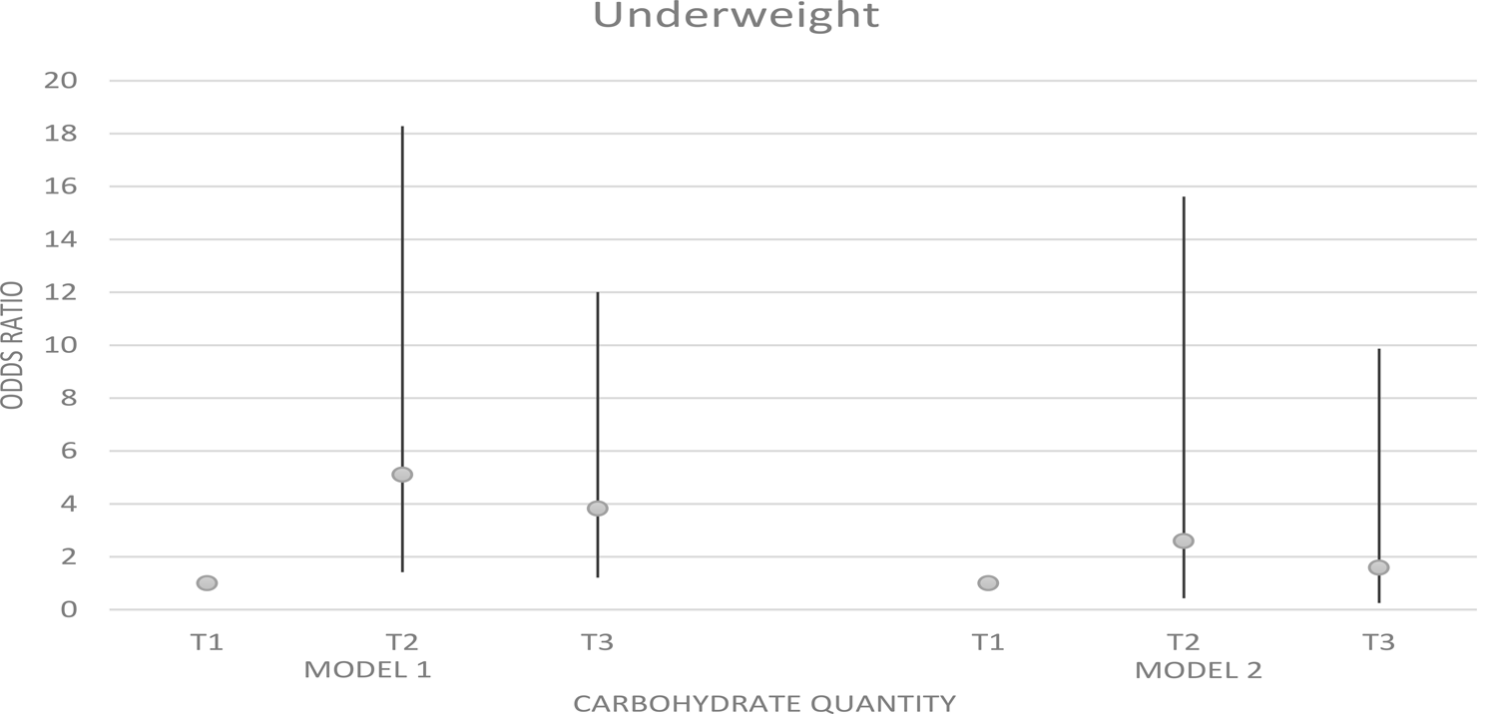
Odds ratio and 95% confidence interval for underweight in tertiles of carbohydrate quantity. Model 1: crude. Model 2: Adjusted for age, supplement use, parents’ smoking, physical activity, and socio-economic status

**Fig. 2:**
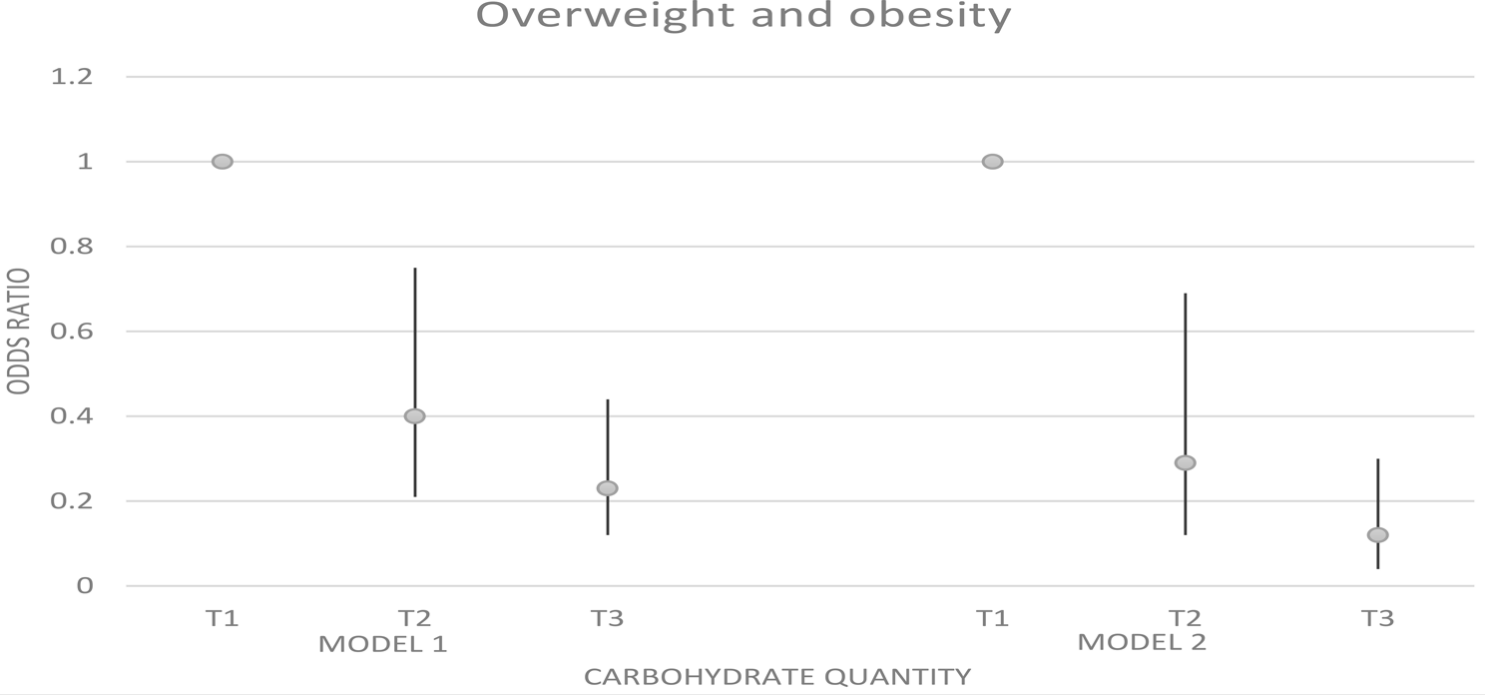
Odds ratio and 95% confidence interval for overweight and obesity in tertiles of carbohydrate quantity. Model 1: crude. Model 2: Adjusted for age, supplement use, parents’ smoking, physical activity, and socio-economic status

Figures 1 and 2 represent the odds ratio and 95% confidence interval for weight disorders in tertiles of carbohydrate intake. We observed a significant association between carbohydrates and overweight and obesity (OR: 0.12; 95% CI: 0.04–0.30; p trend < 0.001). A similar association was detected between carbohydrate intake and underweight before adjusting for confounders (OR: 3.82; 95% CI: 1.21–12.01; p trend = 0.009), But after adjustment, there was no significant association between underweight and carbohydrate intake (p trend = 0.065).

### Odds ratio and 95% confidence interval for weight disorders in tertiles of carbohydrate quality

**Fig. 3:**
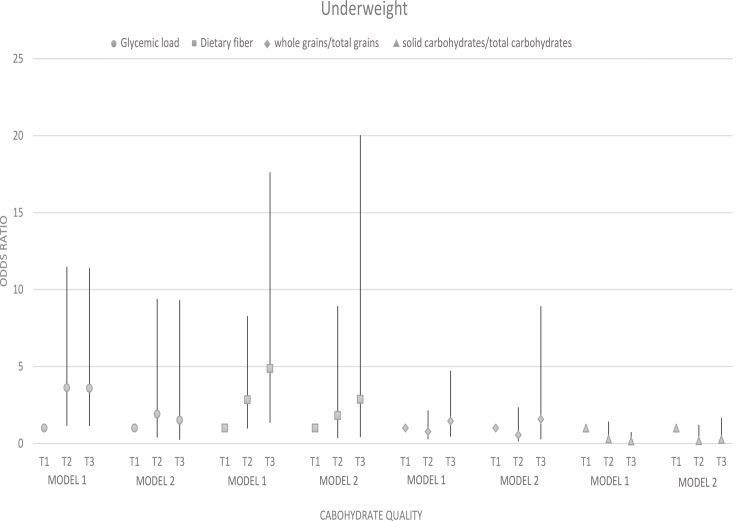
Odds ratio and 95% confidence interval for underweight in tertiles of carbohydrate quality. Model 1: crude. Model 2: Adjusted for age, supplement use, parents’ smoking, physical activity, and socio-economic status

**Fig. 4:**
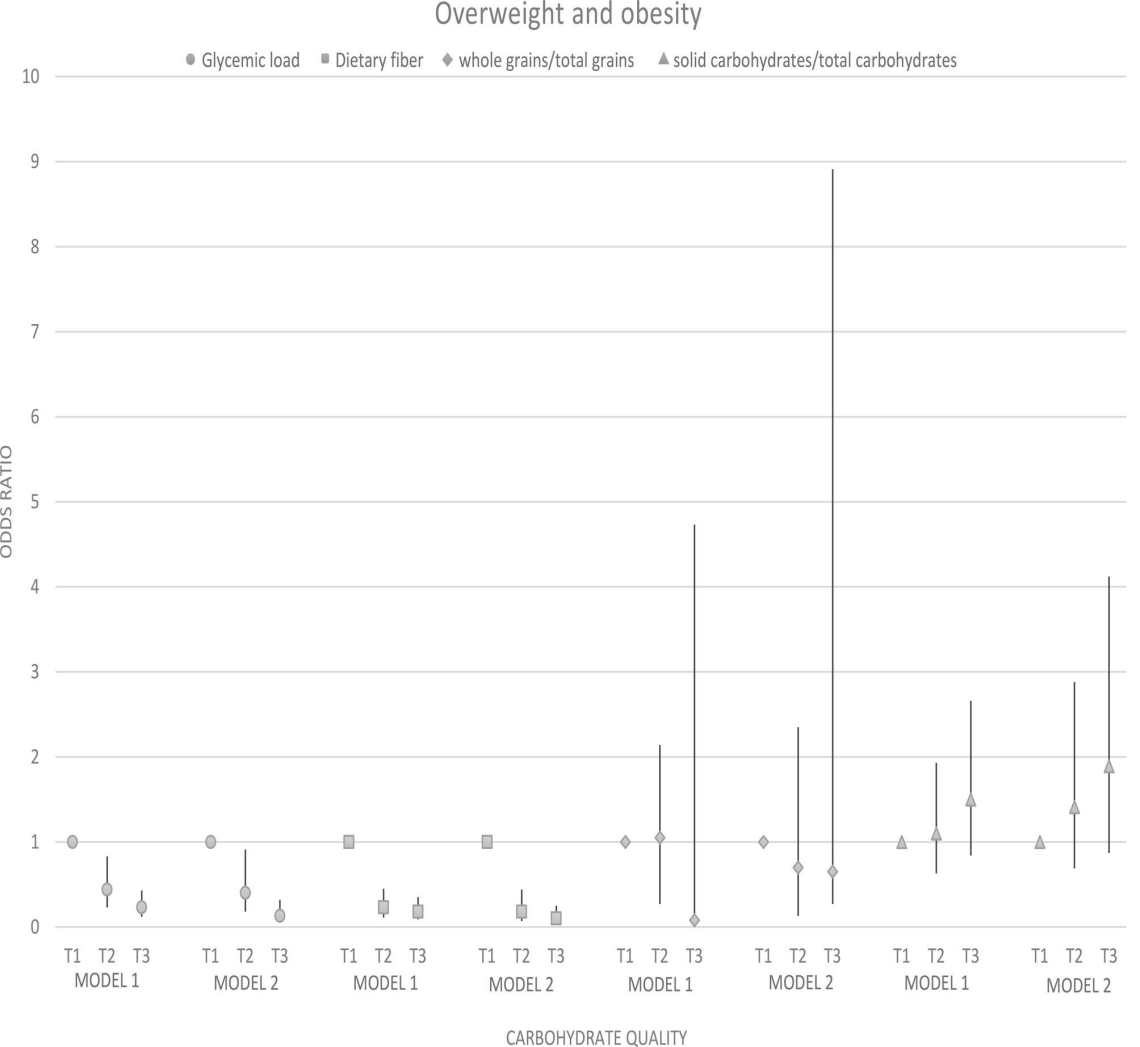
Odds ratio and 95% confidence interval for overweight and obesity in tertiles of carbohydrate quality. Model 1: crude. Model 2: Adjusted for age, supplement use, parents’ smoking, physical activity, and socio-economic status

There was a significant association between glycemic load and underweight (OR: 3.58; 95% CI: 1.13–11.38; p trend = 0.016) and overweight and obesity (OR: 0.23; 95% CI: 0.12–0.43; p trend < 0.001), although, after adjustment, this association disappeared for underweight (p trend = 0.084). A significant association was found between dietary fiber intake and underweight (OR: 4.84; 95% CI: 1.34–17.62; p trend = 0.008) and overweight and obesity (OR: 0.18; 95% CI: 0.09–0.35; p trend = 0.0001) before adjustment. However, the association between dietary fiber intake and being underweight did not remain significant (p trend = 0.065). There was no evidence of a significant association between whole grains/total grains and solid carbohydrates/total carbohydrates and underweight, overweight, and obesity (*p* ≥ 0.05) (Figs. 3 and 4).

## Discussion

The present study examined the association between the quality and quantity of carbohydrate intake with selected anthropometric indices among primary school girls in Kerman. The findings showed a positive association between carbohydrate quantity, glycemic load, and fiber intake, with MUAC, BAZ, and HAZ. We found an inverse association between BAZ and the ratio of solid carbohydrates to total carbohydrates.

The results of this study indicated that the anthropometric parameters including MUAC, BAZ, and HAZ increased with the consumption of carbohydrates. Previously, a study found that HAZ was positively associated with energy intake, protein, carbohydrate, and fat [30]. Also, Claessen et al. found a positive association between a high-carbohydrate diet and waist circumference [31]. Moreover, Demol et al. [32] and Krebs et al. [33] found that the BMI z-score decreased by an average of 0.25 after the low-carb diet. However, other research evaluating the effect of carbohydrate quantity on anthropometric parameters has contradicted our findings. In a clinical trial study on overweight/obese girls aged 9 to 14 years, comparing the effectiveness of a diet with moderate carbohydrate restriction (42%) to a standard carbohydrate diet (55%) on weight loss, no significant difference was seen between weight loss in groups [34]. Another study investigated the association of carbohydrate intake and its subgroups with body composition and metabolic health among 3573 children aged 1–10 years in the Netherlands; no association was found between the intake of carbohydrates and its subgroups with body mass index or body composition in children. In this study, the primary sources of total carbohydrate intake, as well as monosaccharides and disaccharides, were bread and breakfast cereals rather than sugar-containing beverages, which could explain why total carbohydrate or sugar intake was not associated with body composition and inconsistent results with our study [15].

The direct association between glycemic load with anthropometric parameters (MUAC, BAZ, and HAZ) and risk of overweight and obesity in the present study is parallel to the results of a cross-sectional study in the UK which showed a positive association between dietary GL and a higher risk of overweight in children and a higher risk of central obesity (but not being overweight) in adolescents [35]. Similarly, a review suggested that a low-glycemic load diet with a preference for low-glycemic index foods may improve weight-loss maintenance [36]. Hong et al. showed that dietary GI and GL adjusted for energy had no significant correlation with anthropometric data in high school girls living in Seoul [37]. Also, in another study, Cheng et al. found no association between GI, GL, dietary fiber, and whole grain consumption in healthy adolescents and increased body fat percentage or BMI during puberty [38]. In a study by Mendez et al., GL had a negative association with BMI. However, GI was not associated with BMI in any model [39]. Another study by Koksal et al. on the relationship between GI and GL of the diet with body weight, height, body mass index, and waist circumference in adolescents aged 14–18 showed that dietary GI had a positive correlation with anthropometric measurements in both sexes. In contrast, GL had an inverse correlation with body weight and height [40]. The results of these studies were the opposite of the results of the current study, which could be due to heterogeneity in dietary GL consumption patterns. Also, the food data collection tool and the study group differed from ours.

We found positive associations between fiber intake and MUAC, BAZ, and HAZ. Unlike our study, most researchers demonstrated an inverse relationship between fiber and anthropometric indicators. In a prospective cohort study, Du et al. found an inverse relationship between dietary fiber intake, especially cereal fiber, with changes in weight and waist circumference [41]. A meta-analysis (2019) that included 58 trials found that consuming fiber from various sources and whole grains (including oats) reduced average body weight [42]. However, a recent EPIC study reported that dietary fiber from fruits and vegetables was not associated with changes in body weight but with significant changes in waist circumference [41]. In a cross-sectional study on Belgian children aged 6–12 y, no significant association was found between fiber intake and measures of body fat [43]. Matthews et al. found an inverse association between the frequency of consumption of grains, nuts, and vegetables and the risk of being overweight [44]. Evidence showed that the effect of fiber on weight depends on the food source and fiber type [45, 46]. In one study, Stevens et al. found an inverse relationship between cellulose, guar, and psyllium and body weight, but no association was found between wheat bran and energy intake or body weight [46]. Moreover, different cooking and preparation methods contribute to differences in energy density and macronutrient composition, which changes the effect of high-fiber food on body weight.

This study reveals a positive correlation between both fiber and glycemic load and anthropometric indicators, which appears to be contradictory since fiber is known to reduce glycemic load. The observed association can be explained by the fact that individuals who consume more fiber also tend to consume more carbohydrates, which in turn affects the glycemic load. Moreover, as mentioned earlier, high-fiber foods have an impact on weight. It is important to note that a food item may have a high fiber content and a low glycemic load, but still lead to an increase in anthropometric indicators due to the type of fiber and cooking method. For instance, a child may consume fried vegetables rich in fiber, which increases the fat content, or opts for foods with simple sugars, both of which have implications for weight management.

We found no association between the ratio of whole grain to total grain and anthropometric indices. However, in a meta-analysis conducted by Ye et al., it was found that individuals who consumed 48 to 80 g of whole grains daily had less weight gain over 8 to 13 years compared to those who never or rarely consumed whole grains [47]. Moreover, in two epidemiological studies by O’Neil et al. [48] and McKeown et al. [49], consumption of whole grain products was negatively associated with BMI and waist circumference in adults. The difference in study population, which consisted of adults, may be the reason for the different results compared to our study.

Our findings showed an inverse association between BAZ and the ratio of solid carbohydrates to total carbohydrates. Jabraeili et al. conducted another study to investigate the relationship between carbohydrate quality including dietary fiber, glycemic index, and the ratio of solid carbohydrates to total carbohydrates and anthropometric indicators. They showed that participants with higher CQI had lower body weight, GI, glycemic load, energy, and macronutrient intake [17]. The population of this study was adult patients, which was different from ours. Santiago et al. [27] found an inverse relationship between CQI and obesity using four dietary fiber indices, the glycemic index, the ratio of whole grains to total grains, and the ratio of solid carbohydrates to total carbohydrates.

The following mechanisms can explain the effect of carbohydrates on anthropometric parameters. Consuming a high amount of carbohydrates or having a high ratio of carbohydrate to fat or protein causes hyperinsulinemia, which causes a redistribution of fuel and moves carbohydrates from metabolically active tissues to adipose tissue and results in weight gain [50, 51]. The underlying mechanisms for the positive association between dietary GL and anthropometric parameters and risk of obesity found in this study could lead to hyperinsulinemia that can lead to less fat oxidation and more carbohydrate oxidation, potentially leading to more fat storage [52, 53]. Moreover, evidence showed that a low GL diet might reduce blood sugar fluctuations, leading to more prolonged satiety and lower energy intake [52–55]. Whether you consume dietary carbohydrate products in liquid or solid form can have a variable impact on how likely you are to become obese. In general, liquid carbohydrates induce less satiety than solid carbohydrates, increasing the tendency for excess energy intake [56, 57]. Furthermore, liquid carbohydrates have a high glycemic index which may lower insulin sensitivity and raise postprandial blood glucose while raising the risk of obesity and overweight [58].

The strengths of our study were that it was the first study that investigated the relationship between the quality and quantity of carbohydrates and the status of weight and height in primary school girls in Kerman City. Therefore, by presenting findings about the quality and quantity of macronutrients consumed by primary school girls, our study can inform interventions to prevent and reduce weight and height disorders in this age group. The 185-item food frequency questionnaire used in this study was designed specifically for the city of Kerman, which covered most of the local foods consumed by our study population. Its validity and reliability were measured in this study. Also, the children’s physical activity questionnaire used in the present study has already been validated. This study has adjusted the effect of several possible confounders (including age, parent’s education, occupation, supplement use, smoking, physical activity, and socio-economic status).

However, this study has some limitations too. First, the study design was cross-sectional, preventing a definitive cause-and-effect relationship between carbohydrate quality and quantity with weight and height status. Another limitation of our study was using FFQ to assess carbohydrate intake, which may underestimate or overestimate absolute intake amounts. Considering the limitations of the present study, it is suggested that in future studies, the relationship between the quality and quantity of carbohydrates with the anthropometric indices in primary school students should be investigated prospectively. In addition, more research should be conducted in other age and population groups due to the different dietary patterns.

## Conclusions

The present study demonstrated a significant positive association between carbohydrate quantity and anthropometric indices (MUAC, BAZ, and HAZ). There was an inverse association between BAZ and the ratio of solid carbohydrates to total carbohydrates. Also, a significant positive association was revealed between anthropometric indices (MUAC, BAZ, and HAZ) and glycemic load and dietary fiber intake.

## Data Availability

The data sets used and analyzed during the current study are available from the corresponding author upon reasonable request.
